# Cholesterol-Modified Anti-Il6 siRNA Reduces the Severity of Acute Lung Injury in Mice

**DOI:** 10.3390/cells13090767

**Published:** 2024-04-30

**Authors:** Ivan V. Chernikov, Irina K. Bachkova, Aleksandra V. Sen’kova, Mariya I. Meschaninova, Innokenty A. Savin, Valentin V. Vlassov, Marina A. Zenkova, Elena L. Chernolovskaya

**Affiliations:** 1 Institute of Chemical Biology and Fundamental Medicine, Siberian Branch of the Russian Academy of Sciences, Acad. Lavrentiev Ave. 8, 630090 Novosibirsk, Russia; chernikovivanv@gmail.com (I.V.C.); i.bachkova@g.nsu.ru (I.K.B.); senkova_av@niboch.nsc.ru (A.V.S.); mesch@niboch.nsc.ru (M.I.M.); savin_ia@niboch.nsc.ru (I.A.S.); marzen@niboch.nsc.ru (M.A.Z.); 2 Faculty of Natural Sciences, Novosibirsk State University, Pirogova Str., 1, 630090 Novosibirsk, Russia

**Keywords:** acute lung injury, inflammation, chemically modified siRNA, IL6, LPS

## Abstract

Small interfering RNA (siRNA) holds significant therapeutic potential by silencing target genes through RNA interference. Current clinical applications of siRNA have been primarily limited to liver diseases, while achievements in delivery methods are expanding their applications to various organs, including the lungs. Cholesterol-conjugated siRNA emerges as a promising delivery approach due to its low toxicity and high efficiency. This study focuses on developing a cholesterol-conjugated anti-*Il6* siRNA and the evaluation of its potency for the potential treatment of inflammatory diseases using the example of acute lung injury (ALI). The biological activities of different *Il6*-targeted siRNAs containing chemical modifications were evaluated in J774 cells in vitro. The lead cholesterol-conjugated anti-*Il6* siRNA after intranasal instillation demonstrated dose-dependent therapeutic effects in a mouse model of ALI induced by lipopolysaccharide (LPS). The treatment significantly reduced *Il6* mRNA levels, inflammatory cell infiltration, and the severity of lung inflammation. IL6 silencing by cholesterol-conjugated siRNA proves to be a promising strategy for treating inflammatory diseases, with potential applications beyond the lungs.

## 1. Introduction

Small interfering RNAs (siRNAs) silence the expression of the target genes by inducing RNA interference (RNAi) [1]. The catalytic sequence-specific silencing activity of siRNA provides high efficiency and selectivity of their action, which allows them to be considered as a new class of drugs for the therapy of diseases that are not treatable by small molecules or monoclonal antibodies [2]. Currently, the clinical use of siRNA is limited to liver diseases; however, methods for delivering RNA to other organs, including the brain and spinal cord [3,4], kidneys [5,6], spleen [7], and lungs [3,8], are currently under development. The employment of siRNA covalent conjugates with transport ligands is the most promising among siRNA delivery methods due to their low toxicity and high delivery efficiency [9]. Four of the five siRNA-based drugs already approved for clinical use are siRNA conjugates, and they demonstrate safe toxicity and immunogenicity profiles [10,11,12,13,14]. The lungs are important target organs for siRNA therapy since they are involved in the development of life-threatening diseases. On the other hand, non-invasive local delivery to the lungs could be used to reduce the incidence of systemic side effects and provide effective accumulation of the drug. The use of cholesterol-conjugated siRNA for this purpose seems particularly promising since it can retard organs after local delivery and excludes the use of delivery vehicles to avoid additional lung injury [15,16,17]. Therefore, in this work, we used intranasal administration of cholesterol-containing siRNA conjugates to block target gene expression in the lungs.

Inflammatory diseases affect, according to various estimates, 5–9% of the world population [18,19], and their incidence increases every year [20]. Inflammation is associated with reduced life expectancy [19], as well as an elevated risk of cancer [21]. Existing drugs for the treatment of inflammation are characterized by side effects, including toxicity, immunogenicity, and an increased risk of developing infectious diseases [22,23]. In addition, not all patients respond to existing therapy, and drug resistance may develop over time [22,24].

Acute lung injury (ALI), or its more severe manifestation, acute respiratory distress syndrome (ARDS), is a clinical syndrome characterized by damage to the vascular endothelium and alveolar epithelium, which leads to interstitial and pulmonary edema and, ultimately, alveolar collapse [25]. An important role in increasing the permeability of the alveolar–capillary barrier is played by neutrophils that migrate into the lung, secreting pro-inflammatory and pro-apoptotic mediators that damage neighboring cells [26]. An estimated incidence of ALI/ARDS is 64.2 cases per 100,000 person-years, and the mortality rate is 29–42% [26,27]. ALI treatment is not adequately addressed by available therapy and represents an unmet medical need.

We chose interleukin-6 (*Il6*) as a target gene, which is one of the master regulators of inflammatory processes [28], facilitating neutrophil recruitment to the lungs [29]. A decrease in its level is associated with a weakening of ALI symptoms [30]. Thus, in this work, we developed an anti-*Il6* siRNA, conjugated it with cholesterol, validated its silencing activity in vitro, and examined its anti-inflammatory properties in the ALI mouse model.

## 2. Materials and Methods

### 2.1. Synthesis of siRNAs

The anti-*Il6* siRNA sequences are described in Table 1. The control siRNA (siSCR) exhibits no discernible resemblance to any known mouse, rat, or human mRNA sequence. Oligoribonucleotides and their analogs were prepared by the phosphoramidite method on an automatic ASM-800 synthesizer (Biosset, Novosibirsk, Russia). The synthesis employed CPG polymeric carriers with an attached first nucleoside (Glen Research, Sterling, VA, USA), 2′-*O*-TBDMS-protected, 2′-F-, and 2′-O-Me-ribophosphoramidites. Sulfurizing Reagent II (Glen Research, Sterling, VA, USA) was used to introduce phosphothioate linkages. Activation of the free 5′-hydroxyl group of a protected polymer-bound oligonucleotide with N,N’-disuccimidyl carbonate (Acros Organics, Geel, Belgium) was used to synthesize siRNA conjugates with a cholesterol residue and a hexa-methylene linker at the 5′-end. This was followed by an interaction with cholesteryl-6-aminohexylcarbamate, using [31] as an analogy. The target products were isolated using preparative gel electrophoresis in 15% polyacrylamide gel (PAAG) under denaturing conditions after routine deprotection. The products were then eluted using a 0.3 M NaClO_4_ solution. The isolated products were precipitated using a 2% NaClO_4_ solution in acetone after being desalted on a Sep-Pac C18 cartridge (Waters, Milford, MA, USA) or Amicon Ultra 3K (Millipore, Burlington, MA, USA). Equimolar amounts of the sense and antisense siRNA strands were incubated for five minutes at 90 °C in 30 mM HEPES-KOH (pH 7.4), 100 mM potassium acetate, and 2 mM magnesium acetate in order to produce duplexes. The strands hybridized for one hour at a gradually decreasing temperature, and the duplexes were then kept at –20 °C.

### 2.2. Cell Culture

The J774 macrophage cell line was acquired from the Russian Cell Culture Collection (Institute of Cytology, RAS, St. Petersburg, Russia). Dulbecco’s modified Eagle’s medium (DMEM) (Sigma-Aldrich Inc., St. Louis, MO, USA) supplemented with 10% heat-inactivated fetal bovine serum (BioloT, St. Petersburg, Russia) and an antibiotic–antimycotic solution (100 U/mL penicillin, 100 μg/mL streptomycin, 0.25 μg/mL amphotericin) were used for cell culture at 37 °C in a humidified 5% CO_2_-containing air atmosphere (hereafter standard conditions).

### 2.3. Mice

Balb/C mice, aged 6–8 weeks, with an average weight of 20–22 g, were procured from the Institute of Chemical Biology and Fundamental Medicine SB RAS Vivarium (Novosibirsk, Russia). The mice were kept in regular daylight settings in plastic cages with three to six animals per cage. Food and water were available at all times. Experiments were conducted in compliance with the European Communities Council Directive 86/609/CEE. The committee on the ethics of animal experiments of the Siberian Branch Administration of the Russian Academy of Sciences (Novosibirsk, Russia) accepted the experimental protocols (protocol No. 56 from 10 August 2019).

### 2.4. Transfection of siRNA

J774 cells in the exponential phase of growth were plated at a density of 1.5 × 105 cells/well in 24-well plates one day before the experiment. The growth medium was changed with a fresh, serum-free DMEM after 24 h. Following the manufacturer’s instructions, 2 μL of Lipofectamine 2000 (Invitrogen, Waltham, MA, USA) was used per well to transfect the cells with siRNAs (1–100 nM). To avoid overgrowth, cells were replated two days post-transfection. Then, 4 days post-transfection, LPS (final concentration: 1 nM) was added to the cells. Six hours after the addition of LPS, total RNA was isolated from the cells using a kit for RNA isolation, Namagp100 (Biolabmix, Novosibirsk, Russia), and an Auto-Pure 96 automatic nucleic acid isolation and purification system (Allsheng, Hangzhou, China), according to the manufacturer’s protocol. RT-qPCR was performed using M-MuLV-RH revertase and BioMaster HS-qPCR (Biolabmix, Novosibirsk, Russia). Using Hprt mRNA as an internal reference, the amount of Il6 mRNA was defined. The primers and probes listed below were utilized to measure the mRNA levels of the genes:

Il6_F: 5′-AAACCGCTATGAAGTTCCTCTC-3′

IL6_Probe: 5′-((5,6)-FAM)-TTGTCACCAGCATCAGTCCCAAGA-3′-BHQ1

IL6_R: 5′-GTGGTATCCTCTGTGAAGTCTC-3′

Hprt_F: 5′-CCCCAAAATGGTTAAGGTTGC-3′

Hprt_Probe: 5′-((5,6)-ROX)-CTTGCTGGTGAAAAGGACCT-3′-BHQ2

Hprt_R: 5′-AACAAAGTCTGGCCTGTATCC-3′

Data processing was carried out using Bio-Rad CFX Manager 3.1 software (Bio-Rad Laboratories Inc., Hercules, CA, USA).

### 2.5. LPS-Induced Acute Lung Injury (ALI)

Under isoflurane anesthesia, mice (*n* = 3–5 per group) were challenged with LPS (10 µg per mouse, 055:B5, Sigma-Aldrich, USA) via intranasal (i.n.) instillations. Ch-siIL6PS were administered i.n. 4 days before ALI induction. Six hours after the induction of lung inflammation, the mice were euthanized, and 1 mL of ice-cold saline buffer was used to lavage the lungs.

The 20 µL of collected bronchoalveolar lavage (BAL) fluids were processed for cell counting. A total of 20 µL BAL fluids were incubated for 7 min after the addition of a 400 µL solution with 150 mM NH_4_Cl, 10 mM NaHCO_3_, and 0.1 mM EDTA (pH 7.5). Then, the solution was centrifuged for 5 min at 500 g at room temperature, the supernatant was removed, and cells were counted in 30 µL of saline solution. A total of 50 µL of bronchoalveolar cell suspension were put onto slides, stained with azur-eosin using the Romanovsky–Giemsa method, and observed under a microscope to ascertain the differential leukocyte counts. Results were presented as the total leukocyte count (×105 cells/mL) and the percentages of the granulocyte, lymphocyte, and monocyte subpopulations (%).

The rest of the BAL fluids were centrifuged (1600 rpm, 10 min, 4 °C), the supernatant was removed, and RNA isolation and RT-qPCR were carried out as described above.

### 2.6. Histology

Lung specimens were fixed in 10% neutral-buffered formalin, dried in escalating ethanols and xylols, and embedded in HISTOMIX paraffin (BioVitrum, St. Petersburg, Russia) for the histological investigation. A hematoxylin and eosin assay was used to stain paraffin sections (up to 5 µm) that had been sliced using a Thermo Fisher Scientific Microm HM 355 S microtome (Waltham, MA, USA). Using an Axiostar Plus microscope with an Axiocam MRc5 digital camera (Zeiss, Oberkochen, Germany) at ×200 magnification, all the images were examined and scanned.

The intensity of inflammatory infiltration in the lung tissue was assessed using a semi-quantitative approach; the intensity of inflammatory infiltration is represented by a number: 0 indicates no inflammatory infiltration, 1 denotes low intensity, 2 represents moderate intensity, and 3 denotes high intensity. Morphometric analysis of lung sections included a quantitative assessment of alveolar septa’s volume densities (Vv, %) outside the foci of inflammatory infiltration, reflecting interstitial edema, and was carried out with a counting grid that had 100 test points in a testing area that matched 3.2 × 10^6^ μm^2^. The quantification was performed at a magnification of ×200 in five test fields for each lung sample; the number of samples studied was from two to four for each experimental group; thus, analysis of 10–20 random fields was conducted for every experimental group.

### 2.7. Statistical Analysis

The variables were expressed as the mean ± standard deviation (SD) or standard error of the mean (SEM). The data were processed using the Student’s *t*-test. The data obtained in vivo were statistically processed using a two-way ANOVA followed by Bonferroni’s post hoc test. The differences between the values are considered statistically significant at *p* < 0.05. The package STATISTICA, version 10.0, was used for analysis.

## 3. Results

### 3.1. Silencing Activities of Anti-Il6 siRNAs In Vitro

**Figure 1:**
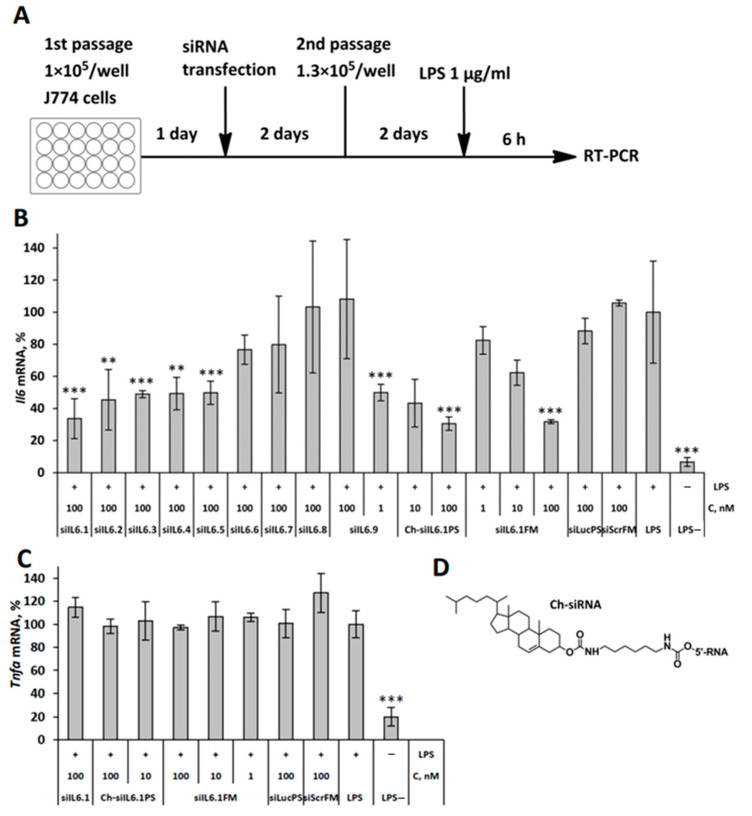
Silencing activity of anti-*Il6* siRNAs and their cholesterol derivatives in J774 cells. (**A**) Experimental setup. Relative *Il6* (**B**) and *Tnfa* (**C**) mRNA levels in J774 cells after LPS stimulation (1 μg/mL) siRNA transfection were carried out with Lipofectamine 2000. Mean values (±SD) and statistical significance of differences from LPS-treated cells (** *p* < 0.01, *** *p* < 0.01), calculated from the results of three independent experiments, are shown in the figure. (**D**) Schematic structure of the cholesterol conjugate.

We designed nine 2′OMe selectively modified siRNAs targeting *Il6* to silence its mRNA level (Table 1). After RNA synthesis and RNA duplex hybridization, we examined the silencing activity of the siRNAs in J774 cells using Lipofectamine 2000 as a transfection agent (Figure 1A).

The level of *Il6* mRNA decreased significantly after transfection of siRNAs siIL6.1–siIL6.5 with 50–66% efficacy; other siRNAs were less active (Figure 1B). The sequence of the most effective siRNA (siIL6.1) was used for the design of siRNA for in vivo experiments: 2′F, 2′OMe modifications were introduced according to [32] to improve nuclease resistance and silencing activity (siIL6.1FM). Further, siIL6FM was conjugated with cholesterol to provide carrier-free delivery of siRNA to target cells and equipped with additional phosphorothioate modifications (PS), resulting in Ch-siIL6.1PS, since nuclease resistance is more important to maintain the integrity of siRNA when delivered without a carrier in vivo than under transfection in vitro [33,34]. A total of 100 nM siIL6.1FM and Ch-siIL6.1PS reduced *Il6* mRNA levels after transfection with Lipofectamine 2000 with 69% and 68% efficacy, which is comparable with the action of selectively modified siIL6.1. However, Ch-siIL6.1PS was more active at lower concentrations than siIL6.1FM under transfection, with IC_50_ values of 1 and 15 nM, respectively (Figure 1B). The decrease in siRNA activity upon the attachment of cholesterol is likely related to the steric block of siRNA interaction with RNAi machinery and has been observed previously [33]. No effect of siIL6.1, Ch-siIL6.1PS, or siIL6.1FM on the level of mRNA of another cytokine activated by LPS, TNF-α, was detected, which confirms the specificity of the action (Figure 1C). Since Ch-siIL6.1PS efficiently decreased *Il6* mRNA levels, we used this conjugate in vivo.

### 3.2. Ch-siIL6.1PS Silences Il6 mRNA Level and Reduces the Severity of Acute Lung Injury in Mice

**Figure 2:**
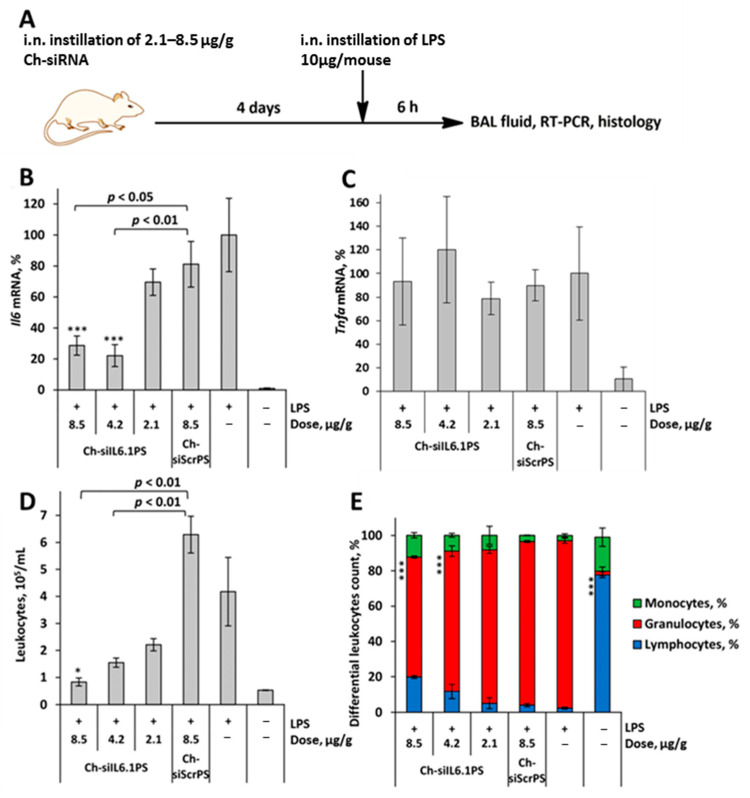
Biological effects of cholesterol-modified anti-Il6 siRNA (Ch-siIL6.1PS) on the development of ALI. (**A**) Experimental setup. (**B**) Il6 mRNA levels in BAL fluid cells. (**C**) *Tnfa* mRNA levels in BAL fluid cells. (**D**) Total and (**E**) differential leukocyte counts in the BAL fluid of healthy (LPS-) and LPS-challenged mice after Ch-siIL6.1PS administration and without treatment. Data are presented as mean ± standard error of the mean, *n* = 3–4, the difference from LPS-challenged mice: * *p* < 0.05, *** *p* < 0.001.

We used the acute lung injury (ALI) model in order to evaluate the significance of modulating the *Il6* mRNA level in alleviating lung tissue damage and determine the effectiveness of the obtained Ch-siIL6.1PS in vivo. Ch-siIL6.1PS was instilled intranasally (i.n.) 4 days before ALI induction by i.n. administration of LPS. The scheme including siRNA pretreatment was chosen because the dynamics of silencing caused by siRNA cholesterol conjugates develop more slowly than the activation of cytokine expression. The control and experimental animals were euthanized 6 h after ALI induction, and the *Il6* mRNA level in bronchoalveolar lavage (BAL) fluid cells and pro-inflammatory parameters in BAL fluid and in lung tissue were analyzed (Figure 2A).

The time intervals were selected based on our previously obtained data on the dynamics of activation of IL6 synthesis during LPS-mediated ALI [35]. Data showed that the *Il6* mRNA level increased by two orders of magnitude in LPS-challenged mice compared to the untreated group (Figure 2B). Instillation of Ch-siIL6.1PS at doses 2.1, 4.2, and 8.5 μg/g decreased *Il6* mRNA level in BAL fluid cells of LPS-challenged mice by 30, 71, and 78%, respectively (*p* < 0.001 for Ch-siIL6.1PS at 8.5 and 4.2 μg/g vs. LPS-challenged group; *p* < 0.05 and *p* < 0.01 for Ch-siIL6.1PS at 8.5 and 4.2 μg/g vs. Ch-siScrPS at dose 8.5 μg/g, respectively). No effect of Ch-siIL6.1PS or siScrPS on the level of *Tnfa* mRNA was detected, which confirms the specificity of the action (Figure 2C).

**Figure 3:**
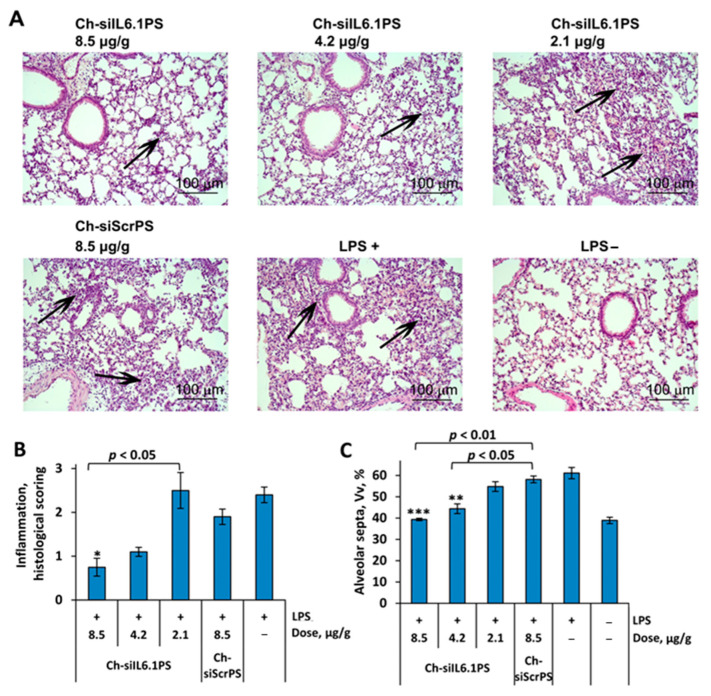
Ch-siIL6.1PS effectively suppresses LPS-induced lung inflammation in vivo. (**A**) Representative histological images of lung tissue from ALI mice. Hematoxylin and eosin staining. Original magnification: ×200. Black arrows indicate inflammatory infiltration in the lung tissue. (**B**) The intensity of inflammatory infiltration in the lung tissue of LPS-challenged mice was calculated by the semi-quantitative histological scoring system, where 0 is no inflammatory infiltration, 1 is low intensity of inflammatory infiltration, 2 is moderate intensity of inflammatory infiltration, and 3 is high intensity of inflammatory infiltration. (**C**) The volume densities (Vv, %) of alveolar septa reflect the intensity of interstitial edema in the lung tissue of LPS-challenged mice without treatment and after Ch-siIL6.1PS administration. All quantifications were performed in five random fields in each lung sample, forming 10–20 random fields from each experimental group. Data are presented as mean ± standard error of the mean, *n* = 3–5, the difference from LPS-challenged mice: * *p* < 0.05, ** *p* < 0.01, *** *p* < 0.001.

The analysis of inflammatory changes in the respiratory system showed that LPS instillation increased the number of total leukocytes in BAL fluid by 8-fold (Figure 2C), predominantly due to granulocytes, compared with healthy animals (Figure 2D). Histological analysis of lung tissue of LPS-challenged mice revealed inflammatory and destructive changes represented by granulocyte infiltration and exudation, as well as desquamation of the bronchial and alveolar epithelium (Figure 3A,B). Moreover, LPS administration led to a 1.6-fold increase in the volume density of the alveolar septa, reflecting interstitial lung edema as one of the morphological components of ALI compared with healthy mice (Figure 3C).

The administration of Ch-siScrPS had no significant effect on the intensity of inflammatory changes in both BAL fluid and lung tissue of mice with ALI (Figure 2D,E). Pretreatment of LPS-challenged mice with Ch-siIL6.1PS caused marked suppression of inflammatory changes in the respiratory system. As shown in Figure 2C, administration of Ch-siIL6.1PS at a dose of 2.1, 4.2, and 8.5 μg/g diminished the number of total leukocytes in BAL fluid 6 h after ALI induction 1.9, 2.7, and 5-fold compared to the non-treated control and 2.8, 4.1, and 7.6-fold compared to the Ch-siScrPS-treated group, respectively. Analysis of leukocyte subpopulations in the BAL fluid of Ch-siIL6.1PS-treated ALI mice demonstrated their ability to suppress inflammation-associated granulocyte recruitment, especially at a dose of 8.5 μg/g (Figure 2E).

Histological study of the lungs of ALI mice confirmed that administration of Ch-siIL6.1PS at a dose of 4.2 and 8.5 μg/g prevented the development of LPS-induced inflammatory changes in the respiratory system, with a 2.2- and 3.2-fold decrease in the intensity of inflammatory infiltration in the lung tissue compared to the control and a 1.7- and 2.5-fold decrease compared to Ch-siScrPS, respectively (Figure 3B). However, statistically significant differences were detected only for Ch-siIL6.1PS at a dose of 8.5 μg/g. Only residual inflammatory cells around the bronchi of experimental mice were detected. Assessing interstitial edema, it was also revealed that treatment of ALI mice with Ch-siIL6.1PS at a dose of 4.2 and 8.5 μg/g led to a 1.4- and 1.6-fold decrease in the volume density of alveolar septa compared to the control and a 1.3- and 1.5-fold decrease compared to Ch-siScrPS, respectively, causing almost normalization of this parameter (Figure 3C). All the identified differences were statistically significant. Histological examination of the lungs of healthy mice not exposed to LPS treated with Ch-siIL6.1PS or Ch-ScrPS at a dose of 8.5 μg/g did not show any changes (Figure S1). Thus, our findings clearly demonstrated that Ch-siIL6.1PS at a dose of 8.5 μg/g effectively prevents the development of LPS-induced ALI.

## 4. Discussion

Acute inflammation is a protective response to infection or tissue damage, but chronic inflammation, on the contrary, can lead to tissue damage and the development of pathological conditions such as fibrosis, autoimmune processes, malignant transformation, and metastasis [25,26]. Corticosteroids are widely used to treat chronic inflammatory diseases, but their effectiveness as an anti-inflammatory drug is limited by steroid drug resistance, which occurs or develops in a significant proportion of patients [36]. Long-term use of corticosteroids, which are required to treat chronic inflammation, can lead to serious side effects, causing immunosuppression, hypertension, diabetes, and adrenal dysfunction manifested by excess cortisol production [37]. The use of specific antibodies to combat inflammation has shown promising results, but the technology is not yet free from unwanted side effects [38]. So, for example, antidrug antibodies were detected in 53% of patients taking adalimumab (a fully humanized IgG1 anti-*TNFa* monoclonal antibody [22]), and high levels of antidrug antibodies were shown to lead to a reduced clinical response [39]. siRNA-based drugs are much less toxic compared to small-molecule drugs, and they mostly do not cause an immune response to siRNA as antibodies do [11,12,13,14]. Antidrug antibodies were detected only in 0.9%, 6.0%, 1.7%, and 2.5% of patients receiving siRNA-based drugs givosiran, lumasiran, inclisiran, and vutrisiran, respectively, and they did not have a significant effect on PK, PD, efficacy, or safety [40,41,42]. Therefore, the development of drugs based on siRNA conjugates for the treatment of inflammatory diseases that are refractory to standard therapy is promising.

Previously, we demonstrated the possibility of reducing the severity of acute injury in the lungs after intranasal instillation of anti-*Timp1* siRNA complexed with cationic liposomes [35]. We showed that the reduction in inflammation was accompanied by a reduced number of neutrophils in the BAL fluid. We also showed that silencing *Timp1* expression by siRNA led to a significant decrease in *Il6* mRNA level, which can prevent leukocyte chemotaxis into lung tissue. IL6 is a multifunctional cytokine that plays an important role in a wide range of biological processes in various cell types, including tumor cells. There is evidence in the literature that deregulated IL6 expression is associated with tumor progression through inhibition of cancer cell apoptosis, stimulation of angiogenesis, and drug resistance [43]. In chronic inflammation, IL6 plays a detrimental role by promoting the accumulation of mononuclear cells at the site of injury through persistent MCP-1 secretion, angioproliferation, and inhibition of T-cell apoptosis. Circulating levels of IL6 are elevated in a number of inflammatory diseases, including rheumatoid arthritis, systemic lupus erythematosus, and Crohn’s disease [7,44]. Blocking IL6 signaling is a potential strategy for treating cancers characterized by pathological overproduction of IL6.

In this study, we directly silenced *Il6* and found that this approach was effective in reducing the severity of ALI. We showed that cholesterol-conjugated anti-*Il6* siRNA, selected based on the results of preliminary in vitro screening, has a dose-dependent therapeutic effect in LPS-induced ALI, reducing the number of cells in BAL fluid and the level of *Il6* mRNA in them, as well as reducing the severity of inflammatory and edema changes in the lungs. The treatment of mice with anti-*Il6* siRNA not only significantly changed the proportion of neutrophils in BAL but also reduced the absolute number of leukocytes compared with LPS-challenged mice, which was not achieved by the anti-*Timp1* siRNA treatment. The relative change in inflammatory processes evaluated by histological scoring also showed positive trends: 2.5 and 3.2 folds for anti-*Timp1* and anti-*Il6* siRNAs, respectively [35]. However, these differences in therapy efficacy may also be due to the chosen targets as well as to different delivery methods and target cells. In a previous study [35], anti-*Timp1* delivery to the target lung cells was performed using cationic lipids. In this study, we delivered anti-*Il6* siRNA by covalent attachment to cholesterol in order to deliver it to alveolar macrophages. It should be noted that we also tried delivering anti-*Il6* siRNA with cationic lipids; however, our preliminary data showed that delivery with a cholesterol conjugate was 44% more effective in reducing the level of *Il6* mRNA in BAL fluid cells than siRNA/liposome complex [data not presented].

Cytokines, including IL6, are quite difficult targets for regulation by siRNAs because their expression increases rapidly and produces a significant amount of mRNA, the translation products of which are secreted from the cell and have an effect on surrounding tissues or even the entire organism. In this work, we applied Ch-siIL6.1PS to prevent ALI in mice in pretreatment mode; however, it is basically impossible to predict the rapid development of ALI except in cases of epidemic or pathogen exposure. Therefore, the question of whether the developed siRNA can be used in therapeutic regimens during the development of the disease remains open. This may be hampered by the relatively slow development of the silencing effect by cholesterol conjugates of siRNA compared to siRNA/liposome complexes because the conjugates are entrapped in the endosomes [45,46]. On the other hand, the ability to avoid the use of lipid delivery systems, which themselves can have an immunostimulating effect and cause side effects, may increase the safety of the preparation.

Two main factors limit the length of time during which a single administration of siRNA can prevent the development of ALI: the nuclease resistance of siRNA and the lifetime of target cells in the lungs. Since siRNA is fully protected by 2′F or 2′OMe modifications, as well as PS at the ends of the duplex, it can be expected that its nuclease resistance will allow it to cause silencing within several months, as has been documented for a drug with similar modifications in the liver [32,47]. However, the second factor more strongly limits the duration of action of Ch-siIL6.1PS in the lungs after local application. The primary target of siRNA is resident alveolar macrophages, which, in response to LPS, attract macrophages and monocytes from the bloodstream into the lung tissue. Alveolar macrophages reside in the lungs for 1–7 weeks [48]. Therefore, we should not expect the effect of one instillation of Ch-siIL6.1PS to last more than a month. This purely theoretical assessment requires a more detailed experimental evaluation.

Thus, the cholesterol-conjugated anti-*Il6* siRNA showed its high potential for preventing the development of ALI. The findings indicate that IL6 inhibition is a productive strategy to combat inflammatory diseases. It can be assumed that such a strategy could be applied to the treatment of inflammation-associated diseases beyond the lungs, including inflammatory bowel disease [49], hepatic cirrhosis [50], sepsis [51], rheumatoid arthritis [52], psoriasis [53], and others. Moreover, further structural and chemical engineering of the anti-*Il6* siRNA and fine-tuning of the delivery systems may expand the scope of its application.

## Figures and Tables

**Table 1:** Oligoribonucleotide sequences.

Designation	Sequence ^1^
IL6.1.AS	GUUmAUmGCCUmAAGCmAUmAUCmAGUUU
IL6.1.S	ACUmGAUmAUmGCUUmAGGCmAUmAACGC
IL6.2.AS	UmAAGGACCmAAGACCmAUCCmA
IL6.2.S	UmGGAUmGGUCUUmGGUCCUUmAGC
IL6.3.AS	GUCmACUUmGAAAUmGUUmAUmAUmGU
IL6.3.S	AUmAUmAACmAUUUCmAAGUmGACmAC
IL6.4.AS	UUmGGGACmACUmAUUUUmAAUUmAU
IL6.4.S	AAUUmAAAAUmAGUmGUCCCmAACmA
IL6.5.AS	UmGCCUmAAGCmAUmAUCmAGUUUmGU
IL6.5_S	AAACUmGAUmAUmGCUUmAGGCmAUmA
IL6.6.AS	UmGCUmAAUUUmAAAUmAUmGUUUUU
IL6.6.S	AAACmAUmAUUUmAAAUUmAGCmAAU
IL6.7.AS	AGUCGGAGGCUUmAAUmUCmACmA
IL6.7.S	UmGUmAAUUmAAGCCUCCGACUUmG
IL6.8.AS	CUmACCmAAACUmGGAUmAUmAAUCmA
IL6.8.S	AUUmAUmAUCCmAGUUUmGGUmAGCmA
IL6.9.AS	CmAGGAAAUUUmGCCUmAUUmGAAA
IL6.9.S	UCmAAUmAGGCmAAAUUUCCUmGAU
IL6.1FM.AS	GmUmUmAmUmGmCfCmUfAfAfGmCmAmUmAmUmCmAmGmUmUmUm
IL6.1FM.S	AmCfUmGmAmUfAmUmGmCmUmUmAmGfGmCfAmUmAmAmCmGmCm
IL6.1PS.AS	Gm * Um * UmAmUmGmCfCmUfAfAfGmCmAmUmAmUmCmAmGmUm * Um * Um
IL6.1PS.S	Am * Cf * UmGmAmUfAmUmGmCmUmUmAmGfGmCfAmUmAmAmCm * Gm * Cm
Ch-IL6.1PS.AS	Ch-Gm * Um * UmAmUmGmCfCmUfAfAfGmCmAmUmAmUmCmAmGmUm * Um * Um
LucPS.AS	Cm * Gm * UmUmAmUmUfUmAfUfCfGmGmAmGmUmUmGmCm * Am * Gm
LucPS.S	Gm * Cf * AmAmCmUfCmCmGmAmUmAmAmAfUmAfAmCmGm * Cm * Gm
ScrFM.AS	AmAfUmAmUmCfUmGmCmUmCmUmUmCfAmUfGmCmGmGmGm
ScrFM.S	CmGmCmAmUmGmAfAmGfAfGfCmAmGmAmUmAmUmUmCmGm
ScrPS.AS	Am * Af * UmAmUmCfUmGmCmUmCmUmUmCfAmUfGmCmGm * Gm * Gm
Ch-ScrPS.S	Ch-Cm * Gm * CmAmUmGmAfAmGfAfGfCmAmGmAmUmAmUmUm * Cm * Gm

^1^ Sequences are in the 5′–3′ direction: Am, Cm, Gm, Um–2′-O-methyl modified; Af, Cf, Gf, Uf–2′-fluoro modified; *—phosphorothioate modification; AS—antisense strand; S—sense strand; Ch—cholesterol residue attached to the 5′ end of the sense strand via a hexamethylenediamine linker.

## Data Availability

Data are contained within the article.

## Supplementary Materials

The following supporting information can be downloaded at: https://www.mdpi.com/article/10.3390/cells13090767/s1, Figure S1: Representative histological images of lung tissue from healthy mice without treatment and after Ch-siIL6.1PS of Ch-siScrPS administration. Hematoxylin and eosin staining. Original magnification ×200.

## References

[1] Dana H., Mahmoodi Chalbatani G., Mahmoodzadeh H., Karimloo R., Rezaiean O., Moradzadeh A., Mehmandoost N., Moazzen F., Mazraeh A., Marmari V. (2017). Molecular Mechanisms and Biological Functions of SiRNA. Int. J. Biomed. Sci..

[2] Hu B., Zhong L., Weng Y., Peng L., Huang Y., Zhao Y., Liang X.J. (2020). Therapeutic SiRNA: State of the Art. Signal Transduct. Target. Ther..

[3] Brown K.M., Nair J.K., Janas M.M., Anglero-Rodriguez Y.I., Dang L.T.H., Peng H., Theile C.S., Castellanos-Rizaldos E., Brown C., Foster D. (2022). Expanding RNAi Therapeutics to Extrahepatic Tissues with Lipophilic Conjugates. Nat. Biotechnol..

[4] Alterman J.F., Godinho B.M.D.C., Hassler M.R., Ferguson C.M., Echeverria D., Sapp E., Haraszti R.A., Coles A.H., Conroy F., Miller R. (2019). A Divalent SiRNA Chemical Scaffold for Potent and Sustained Modulation of Gene Expression throughout the Central Nervous System. Nat. Biotechnol..

[5] Zuckerman J.E., Gale A., Wu P., Ma R., Davis M.E. (2015). SiRNA Delivery to the Glomerular Mesangium Using Polycationic Cyclodextrin Nanoparticles Containing SiRNA. Nucleic Acid Ther..

[6] Thai H.B.D., Kim K.R., Hong K.T., Voitsitskyi T., Lee J.S., Mao C., Ahn D.R. (2020). Kidney-Targeted Cytosolic Delivery of SiRNA Using a Small-Sized Mirror DNA Tetrahedron for Enhanced Potency. ACS Cent. Sci..

[7] Jiang Y., Hardie J., Liu Y., Ray M., Luo X., Das R., Landis R.F., Farkas M.E., Rotello V.M. (2018). Nanocapsule-Mediated Cytosolic SiRNA Delivery for Anti-Inflammatory Treatment. J. Control. Release.

[8] Miller J.B., Kos P., Tieu V., Zhou K., Siegwart D.J. (2018). Development of Cationic Quaternary Ammonium Sulfonamide Amino Lipids for Nucleic Acid Delivery. ACS Appl. Mater. Interfaces.

[9] Chernikov I.V., Vlassov V.V., Chernolovskaya E.L. (2019). Current Development of SiRNA Bioconjugates: From Research to the Clinic. Front. Pharmacol..

[10] Eberle F., Giessler K., Deck C., Heeg K., Peter M., Richert C., Dalpke A.H. (2008). Modifications in Small Interfering RNA That Separate Immunostimulation from RNA Interference. J. Immunol..

[11] Sioud M., Furset G., Cekaite L. (2007). Suppression of Immunostimulatory SiRNA-Driven Innate Immune Activation by 2′-Modified RNAs. Biochem. Biophys. Res. Commun..

[12] Manoharan M., Akinc A., Pandey R.K., Qin J., Hadwiger P., John M., Mills K., Charisse K., Maier M.A., Nechev L. (2011). Unique Gene-Silencing and Structural Properties of 2′-Fluoro-Modified SiRNAs. Angew. Chemie Int. Ed..

[13] Judge A.D., Bola G., Lee A.C.H., MacLachlan I. (2006). Design of Noninflammatory Synthetic SiRNA Mediating Potent Gene Silencing In Vivo. Mol. Ther..

[14] Robbins M., Judge A., Liang L., McClintock K., Yaworski E., MacLachlan I. (2007). 2′-O-Methyl-Modified RNAs Act as TLR7 Antagonists. Mol. Ther..

[15] Chernikov I.V., Gladkikh D.V., Meschaninova M.I., Ven’yaminova A.G., Zenkova M.A., Vlassov V.V., Chernolovskaya E.L. (2017). Cholesterol-Containing Nuclease-Resistant SiRNA Accumulates in Tumors in a Carrier-Free Mode and Silences MDR1 Gene. Mol. Ther.-Nucleic Acids.

[16] Biscans A., Coles A., Haraszti R., Echeverria D., Hassler M., Osborn M., Khvorova A. (2019). Diverse Lipid Conjugates for Functional Extra-Hepatic SiRNA Delivery in Vivo. Nucleic Acids Res..

[17] Chernikov I.V., Meschaninova M.I., Gladkikh D.V., Ven’yaminova A.G., Zenkova M.A., Vlassov V.V., Chernolovskaya E.L. (2021). Interaction of Lipophilic Conjugates of Modified SiRNAs with Hematopoietic Cells In Vitro and In Vivo. Russ. J. Bioorganic Chem..

[18] Cooper G.S., Bynum M.L.K., Somers E.C. (2009). Recent Insights in the Epidemiology of Autoimmune Diseases: Improved Prevalence Estimates and Understanding of Clustering of Diseases. J. Autoimmun..

[19] El-Gabalawy H., Guenther L.C., Bernstein C.N. (2010). Epidemiology of Immune-Mediated Inflammatory Diseases: Incidence, Prevalence, Natural History, and Comorbidities. J. Rheumatol..

[20] Lerner A., Jeremias P., Matthias T. (2015). The World Incidence and Prevalence of Autoimmune Diseases Is Increasing. Int. J. Celiac Dis..

[21] Yasunaga M. (2020). Antibody Therapeutics and Immunoregulation in Cancer and Autoimmune Disease. Semin. Cancer Biol..

[22] Li P., Zheng Y., Chen X. (2017). Drugs for Autoimmune Inflammatory Diseases: From Small Molecule Compounds to Anti-TNF Biologics. Front. Pharmacol..

[23] Connor V. (2011). Anti-TNF Therapies: A Comprehensive Analysis of Adverse Effects Associated with Immunosuppression. Rheumatol. Int..

[24] Yu M.B., Firek A., Langridge W.H.R. (2018). Predicting Methotrexate Resistance in Rheumatoid Arthritis Patients. Inflammopharmacology.

[25] Ragaller M., Richter T. (2010). Acute Lung Injury and Acute Respiratory Distress Syndrome. J. Emergencies Trauma Shock.

[26] Gotzev R., Kenarov P. (2013). Acute Respiratory Distress Syndrome (ARDS). Anaesthesiol. Intensive Care.

[27] Zoulikha M., Xiao Q., Boafo G.F., Sallam M.A., Chen Z., He W. (2022). Pulmonary Delivery of SiRNA against Acute Lung Injury/Acute Respiratory Distress Syndrome. Acta Pharm. Sin. B.

[28] Trovato M., Sciacchitano S., Facciolà A., Valenti A., Visalli G., Di Pietro A. (2021). Interleukin-6 Signalling as a Valuable Cornerstone for Molecular Medicine (Review). Int. J. Mol. Med..

[29] Florentin J., Zhao J., Tai Y.Y., Vasamsetti S.B., O’Neil S.P., Kumar R., Arunkumar A., Watson A., Sembrat J., Bullock G.C. (2021). Interleukin-6 Mediates Neutrophil Mobilization from Bone Marrow in Pulmonary Hypertension. Cell. Mol. Immunol..

[30] Chen I.C., Wang S.C., Chen Y.T., Tseng H.H., Liu P.L., Lin T.C., Wu H.E., Chen Y.R., Tseng Y.H., Hsu J.H. (2021). Corylin Ameliorates Lps-Induced Acute Lung Injury via Suppressing the Mapks and Il-6/Stat3 Signaling Pathways. Pharmaceuticals.

[31] Meschaninova M.I., Novopashina D.S., Semikolenova O.A., Silnikov V.N., Venyaminova A.G. (2019). Novel Convenient Approach to the Solid-Phase Synthesis of Oligonucleotide Conjugates. Molecules.

[32] Foster D.J., Brown C.R., Shaikh S., Trapp C., Schlegel M.K., Qian K., Sehgal A., Rajeev K.G., Jadhav V., Manoharan M. (2018). Advanced SiRNA Designs Further Improve In Vivo Performance of GalNAc-SiRNA Conjugates. Mol. Ther..

[33] Chernikov I.V., Ponomareva U.A., Meschaninova M.I., Bachkova I.K., Teterina A.A., Gladkikh D.V., Savin I.A., Vlassov V.V., Zenkova M.A., Chernolovskaya E.L. (2023). Cholesterol-Conjugated Supramolecular Multimeric SiRNAs: Effect of SiRNA Length on Accumulation and Silencing In Vitro and In Vivo. Nucleic Acid Ther..

[34] Hassler M.R., Turanov A.A., Alterman J.F., Haraszti R.A., Coles A.H., Osborn M.F., Echeverria D., Nikan M., Salomon W.E., Roux L. (2018). Comparison of Partially and Fully Chemically-Modified SiRNA in Conjugate-Mediated Delivery In Vivo. Nucleic Acids Res..

[35] Chernikov I.V., Staroseletz Y.Y., Tatarnikova I.S., Sen’kova A.V., Savin I.A., Markov A.V., Logashenko E.B., Chernolovskaya E.L., Zenkova M.A., Vlassov V.V. (2023). SiRNA-Mediated Timp1 Silencing Inhibited the Inflammatory Phenotype during Acute Lung Injury. Int. J. Mol. Sci..

[36] Liao W., Dong J., Peh H.Y., Tan L.H., Lim K.S., Li L., Wong W.S.F. (2017). Oligonucleotide Therapy for Obstructive and Restrictive Respiratory Diseases. Molecules.

[37] Gupta R., Fonacier L.S. (2016). Adverse Effects of Nonsystemic Steroids (Inhaled, Intranasal, and Cutaneous): A Review of the Literature and Suggested Monitoring Tool. Curr. Allergy Asthma Rep..

[38] Singh R., Chandley P., Rohatgi S. (2023). Recent Advances in the Development of Monoclonal Antibodies and Next-Generation Antibodies. ImmunoHorizons.

[39] van Schouwenburg P.A., Bartelds G.M., Hart M.H., Aarden L., Wolbink G.J., Wouters D. (2010). A Novel Method for the Detection of Antibodies to Adalimumab in the Presence of Drug Reveals “Hidden” Immunogenicity in Rheumatoid Arthritis Patients. J. Immunol. Methods.

[40] Willoughby J.L.S., Chan A., Sehgal A., Butler J.S., Nair J.K., Racie T., Shulga-Morskaya S., Nguyen T., Qian K., Yucius K. (2018). Evaluation of GalNAc-SiRNA Conjugate Activity in Pre-Clinical Animal Models with Reduced Asialoglycoprotein Receptor Expression. Mol. Ther..

[41] Corydon I.J., Fabian-Jessing B.K., Jakobsen T.S., Jørgensen A.C., Jensen E.G., Askou A.L., Aagaard L., Corydon T.J. (2023). 25 Years of Maturation: A Systematic Review of RNAi in the Clinic. Mol. Ther.-Nucleic Acids.

[42] An G. (2024). Pharmacokinetics and Pharmacodynamics of GalNAc-Conjugated SiRNAs. J. Clin. Pharmacol..

[43] Ene C.V., Nicolae I., Geavlete B., Geavlete P., Ene C.D. (2022). IL-6 Signaling Link between Inflammatory Tumor Microenvironment and Prostatic Tumorigenesis. Anal. Cell. Pathol..

[44] Roda G., Chien Ng S., Kotze P.G., Argollo M., Panaccione R., Spinelli A., Kaser A., Peyrin-Biroulet L., Danese S. (2020). Crohn’s Disease. Nat. Rev. Dis. Prim..

[45] Petrova N.S., Chernikov I.V., Meschaninova M.I., Dovydenko I.S., Venyaminova A.G., Zenkova M.A., Vlassov V.V., Chernolovskaya E.L. (2012). Carrier-Free Cellular Uptake and the Gene-Silencing Activity of the Lipophilic SiRNAs Is Strongly Affected by the Length of the Linker between SiRNA and Lipophilic Group. Nucleic Acids Res..

[46] Gilleron J., Querbes W., Zeigerer A., Borodovsky A., Marsico G., Schubert U., Manygoats K., Seifert S., Andree C., Stöter M. (2013). Image-Based Analysis of Lipid Nanoparticle-Mediated SiRNA Delivery, Intracellular Trafficking and Endosomal Escape. Nat. Biotechnol..

[47] Nair J.K., Attarwala H., Sehgal A., Wang Q., Aluri K., Zhang X., Gao M., Liu J., Indrakanti R., Schofield S. (2017). Impact of Enhanced Metabolic Stability on Pharmacokinetics and Pharmacodynamics of GalNAc-SiRNA Conjugates. Nucleic Acids Res..

[48] van oud Alblas A.B., van Furth R. (1979). Origin, Kinetics, and Characteristics in the Normal Macrophages Steady State. J Exp Med..

[49] Veiga N., Goldsmith M., Diesendruck Y., Ramishetti S., Rosenblum D., Elinav E., Behlke M.A., Benhar I., Peer D. (2019). Leukocyte-Specific SiRNA Delivery Revealing IRF8 as a Potential Anti-Inflammatory Target. J. Control. Release.

[50] Zhang J., Shen H., Xu J., Liu L., Tan J., Li M., Xu N., Luo S., Wang J., Yang F. (2020). Liver-Targeted SiRNA Lipid Nanoparticles Treat Hepatic Cirrhosis by Dual Antifibrotic and Anti-Inflammatory Activities. ACS Nano.

[51] Lee J., Son W., Hong J., Song Y., Yang C.S., Kim Y.H. (2021). Down-Regulation of TNF-α via Macrophage-Targeted RNAi System for the Treatment of Acute Inflammatory Sepsis. J. Control. Release.

[52] Liu X., Guo R., Huo S., Chen H., Song Q., Jiang G., Yu Y., Huang J., Xie S., Gao X. (2022). CaP-Based Anti-Inflammatory HIF-1α SiRNA-Encapsulating Nanoparticle for Rheumatoid Arthritis Therapy. J. Control. Release.

[53] Mandal A., Kumbhojkar N., Reilly C., Dharamdasani V., Ukidve A., Ingber D.E., Mitragotri S. (2020). Treatment of Psoriasis with NFKBIZ SiRNA Using Topical Ionic Liquid Formulations. Sci. Adv..

