# Supplementary material for: Cholesterol-Modified Anti-Il6 siRNA Reduces the Severity of Acute Lung Injury in Mice
Source: Cells. 2024 Apr 30;13(9):767. doi: 10.3390/cells13090767 (PMC11083178; doi:10.3390/cells13090767)
Supplement: Supplementary file 1 [file cells-13-00767-s001.zip › cells-2978817-supplementary.pdf]

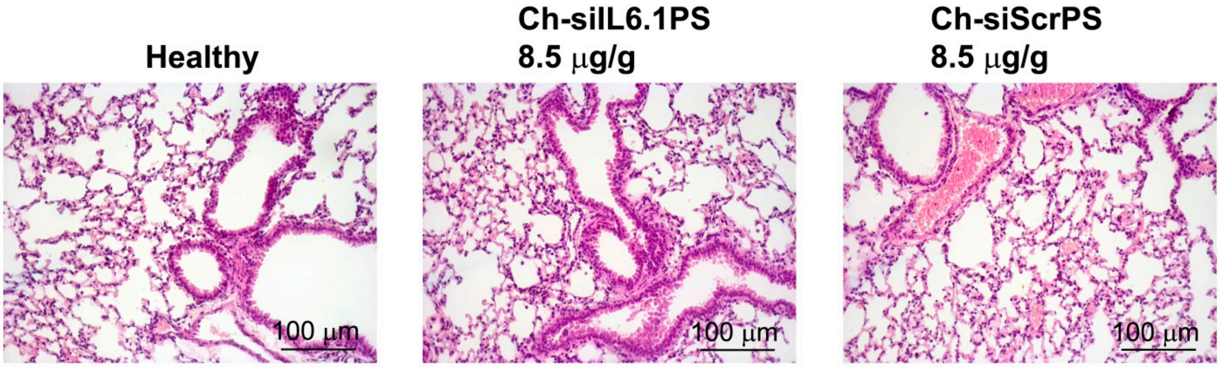

Figure S1. Representative histological images of lung tissue from healthy mice without treatment and after Ch-siIL6.1PS of Ch-siScrPS administration. Hematoxylin and eosin staining. Original magnification ×200.
